# Draft Genome Sequences of Four NDM-1-Producing *Klebsiella pneumoniae* Strains from a Health Care Facility in Northern California

**DOI:** 10.1128/genomeA.00421-15

**Published:** 2015-05-14

**Authors:** Alexander L. Greninger, Ilya Chorny, Susan Knowles, Valerie L. Ng, Vishnu Chaturvedi

**Affiliations:** a Microbial Diseases Laboratory, California Department of Public Health, Richmond, California, USA; b Illumina, Inc., San Diego, California, USA; c Department of Laboratory Medicine and Pathology, Alameda County Medical Center, Oakland, California, USA

## Abstract

We report the draft genome sequences of *Klebsiella pneumoniae* strains from four patients at a northern California health care facility. All strains contained the New Delhi metallo-β-lactamase (NDM1) carbapenemase with extended antibiotic resistance, including resistance to expanded-spectrum cephalosporins, imipenem, ertapenem, and meropenem. NDM gene alignments revealed that the resistance was plasmid encoded.

## GENOME ANNOUNCEMENT

*Klebsiella pneumoniae* is a Gram-negative bacterium that is part of the normal flora of the gastrointestinal tract but can cause serious community- and hospital-acquired infections. Due to the recent evolution of multiple antibiotic resistance genes, *K. pneumoniae* has been denoted as a microbial threat to human health (1, 2). New Delhi metallo-β-lactamase (NDM1) carbapenemase-producing *K. pneumoniae*, originally reported from India, have spread rapidly around the world with many imported strains reported from California (3, 4).

Four *K. pneumoniae* strains came from adult males at a northern California health care facility in summer/fall 2013 and were derived from urine, sputum, a rectal swab, and pigtail drain fluid. The California Department of Public Health initially performed PCR testing for *bla*_KPC_ and *bla*_NDM_.

All four strains were sequenced using Nextera paired-end sequencing and Nextera gel-free mate-pair sequencing and assembly with SPAdes v3.0/v3.1 and annotation via PROKKA v1.10 and NCBI Prokaryotic Annotation Pipeline (5, 6). The Nextera paired-end draft genome assemblies for strains CPH3020, CPH3707, CPH3823, and CPH5262 include 93, 116, 112, and 102 contigs >200 nucleotides measuring a total of 5,468,355 bp, 5,359,393 bp, 5,360,573 bp, 5,351,951 bp, respectively. The *N*_50_s of the strains measure 214,475 bp, 206,548 bp, 206,548 bp, and 172,934 bp with coverages of 70×, 147×, 124×, and 155×, and there are 5,275, 5,182, 5,187, and 5,178 predicted coding sequences (CDS), respectively. Assemblies from the Nextera gel-free mate-pair alone yielded 68, 58, 54, and 59 contigs >200 nucleotides measuring a total of 5,494,465, 5,368,188, 5,371,617, and 5,378,024 bp, respectively. The mate-pair kit had significantly higher *N*_50_s to 2,988,282 bp, 2,989,016 bp, 2,989,036 bp, and 2,989,036 bp with coverages of 65×, 48×, 45×, and 69×, respectively.

Of predicted CDS, 16.2% in strain CPH3020 were annotated as hypothetical proteins, while 15.2% were annotated as hypothetical proteins in the three other strains. GC content ranged from 57.1% for strain CPH3020 to 57.3% for the three other strains. The scaffolds for each genome demonstrated >99.9% nucleotide identity to each other and >99.5% nucleotide identity across >93% the bacterial chromosome of the reference genome *Klebsiella pneumoniae* MGH 78578. Less than 20% of the plasmid sequence of MGH 78578 was covered by scaffolds from these *Klebsiella* strains, with minimal contiguity. All four strains had *bla*_NDM_ resistance genes with 100% nucleotide identity to PMK1-NDM-1 and PittNDM01 NDM-1 genes, with the same flanking 10 kb, including the bleomycin resistance gene, phosphoribosyl anthranilate isomerase, Tn*3* family transposase, and GroES/L (7–9). All strains contained *bla*_OXA-1_ and *bla*_SHV_ and/or *bla*_TEM_ beta-lactamases (10, 11). None of the strains contained the *bla*_KPC_, *bla*_VIM_, or *bla*_OXA-48_ and/or *bla*_OXA-181_ carbapenemase resistance genes nor the *bla*_CTX-15_ extended-spectrum beta-lactamase (12). The alignments of NDM and neighboring genes suggested that the resistance was plasmid encoded, with significant alignments spanning the entirety of the assembled contig to plasmids from NDM-containing strains of *Klebsiella pneumoniae*, *Escherichia coli*, and *Enterobacter hormaechei*. Additionally, the NDM locus had 2× to 3× the coverage of the bacterial chromosome in three of the four strains, suggesting it existed on a different element. The additional ~100 kb in the CDPH3020 genome not present in the other *Klebsiella* strains was entirely derived from the pPMK1-NDM plasmid (9).

### Nucleotide sequence accession numbers. 

This whole-genome shotgun project has been deposited at DDBJ/EMBL/GenBank under the accession numbers JQCW00000000 (3020), JQCX00000000 (3707), JQDX00000000 (3823), and JQDY00000000 (5262) for the paired-end assemblies. The mate-pair assemblies have been deposited at DDBJ/EMBL/GenBank under the accession numbers LAFV00000000 (3020), LAFX00000000 (3707), LAFW00000000 (3823), and LAFU00000000 (5262).
